# Isolation and Genetic Characterization of Canine Parvovirus in a Malayan Tiger

**DOI:** 10.3389/fvets.2021.660046

**Published:** 2021-08-03

**Authors:** Ahmad Nadzri Nur-Farahiyah, Kiven Kumar, Abd Rahaman Yasmin, Abdul Rahman Omar, Siti Nazrina Camalxaman

**Affiliations:** ^1^Department of Veterinary Laboratory Diagnosis, Faculty of Veterinary Medicine, Universiti Putra Malaysia, UPM Serdang, Selangor, Malaysia; ^2^Department of Pathology, Faculty of Medicine and Health Sciences, Universiti Putra Malaysia, UPM Serdang, Selangor, Malaysia; ^3^Laboratory of Vaccines and Biomolecules, Institute of Bioscience, Universiti Putra Malaysia, UPM Serdang, Selangor, Malaysia; ^4^Department of Veterinary Pathology and Microbiology, Faculty of Veterinary Medicine, Universiti Putra Malaysia, UPM Serdang, Selangor, Malaysia; ^5^Department of Medical Laboratory Technology, Faculty of Health Sciences, Universiti Teknologi MARA (UiTM), Selangor, Malaysia

**Keywords:** canine parvovirus, felids, Malayan tiger, full genome, capsid protein, non structural protein, evolution rate

## Abstract

Naïve Felidae in the wild may harbor infectious viruses of importance due to cross-species transmission between the domesticated animals or human–wildlife contact. However, limited information is available on virus shedding or viremia in the captive wild felids, especially in Malaysia. Four infectious viruses of cat, feline herpesvirus (FHV), feline calicivirus (FCV), canine distemper virus (CDV), and canine parvovirus (CPV), were screened in leopards, feral cats, and tigers in Malaysia based on virus isolation in Crandell-Rees feline kidney (CRFK) cells, PCR/RT-PCR, and whole-genome sequencing analysis of the positive isolate. From a total of 36 sera collected, 11 samples showed three consecutive cytopathic effects in the cell culture and were subjected to PCR using specific primers for FHV, FCV, CDV, and CPV. Only one sample from a Malayan tiger was detected positive for CPV. The entire viral genome of CPV (UPM-CPV15/P. tigris jacksoni; GenBank Accession number MW380384) was amplified using the Sanger sequencing approach. Genome sequencing of the isolate revealed 99.13, 98.65, and 98.40% close similarity to CPV-31, CPV-d Cornell #320, and CPV-15 strains, respectively, and classified as CPV-2a. Time-scaled Bayesian Maximum Clade Credibility tree for the non-structural (NS) genes of CPV showed a close relationship to the isolates CPV-CN SD6_2014 and KSU7-SD_2004 from China and USA, respectively, while the capsid gene showed the same ancestor as the FPV-BJ04 strain from China. The higher evolution rate of the capsid protein (CP) (VP 1 and VP2) [1.649 × 10^−5^ (95% HPD: 7.626 × 10^−3^ to 7.440 × 10^−3^)] as compared to the NS gene [1.203 × 10^−4^ (95% HPD: 6.663 × 10^−3^ to 6.593 × 10^−3^)] was observed in the CPV from this study, and fairly higher than other parvovirus species from the *Protoparvovirus* genus. Genome sequencing of the isolated CPV from a Malayan tiger in the present study provides valuable information about the genomic characteristics of captive wild felids, which may add information on the presence of CPV in species other than dogs.

## Introduction

*Parvoviridae* is a family of non-enveloped and single-stranded DNA virus with a 4–6 kb linear genome. It derives its name from the Latin word “parvus,” which means small and widely dispersed in nature (1). *Parvoviridae* family is divided into three subfamilies, namely, *Parvovirinae* (vertebrate viruses), *Densovirinae* (invertebrate viruses), and *Hamaparvovirinae* (found in insects). Members of *Parvoviridae* consist of parvovirus that can infect a diverse range of mammalian, avian, and reptilian hosts. The subfamily *Parvovirinae* are categorized into 10 recognized genera, namely, *Amdoparvovirus, Artiparvovirus, Aveparvovirus, Bocaparvovirus, Copiparvovirus, Dependoparvovirus, Erythroparvovirus, Loriparvovirus, Protoparvovirus*, and *Tetraparvovirus* (2). Among *Protoparvovirus*, examples of virus species are canine parvovirus (CPV) (known as carnivore protoparvovirus 1), feline panleukopenia virus (FPV) (known as carnivore protoparvovirus 1), porcine parvovirus (PPV) (known as ungulate parvovirus 1), rodent protoparvovirus, rodent protoparvovirus 2 (rat parvovirus 1), mink enteritis virus (MEV), and Kilham's rat virus (KRV) (3).

One such important veterinary pathogen known to infect carnivore species is the CPV, a pathogenic agent that causes severe haemorrhagic gastroenteritis in dogs. It emerged during the late 1970's in the canine population (4). Currently, there are three antigenic types of CPV-2 named CPV-2a,−2b, and−2c. The CPV-2 is considered to be derived from FPV of domestic cats (5). As a result of adaptation to feline transferrin receptor, CPV variants are able to infect not only canines but also felines (6, 7). Although, these antigenic variants occur negligibly, they remain important because they can infect cats (8–11), while remaining a threat to dogs. The CPV genome consists of two open reading frames (ORFs) that encode for non-structural proteins (NS1 and NS2) and structural proteins (VP1 and VP2) (12).

Currently, in Malaysia, abundant studies have been exploring the prevalence of common viral pathogens of domesticated cats and dogs, such as feline herpesvirus (FHV), feline calicivirus (FCV), canine distemper virus (CDV) and CPV (13, 14). However, there are limited data on the viremia status of those viruses in the captive wild felids. As felid species are also susceptible to viral infections, this study was performed to investigate the potentially important viruses in diverse species of felids, namely, leopards, feral cats, and tigers in Malaysia from archive sera samples. Consecutively, the study was also attempted to characterize the isolated virus *via* full-genome sequencing analysis.

## Materials and Methods

### Screening of Viruses

Sera from 36 unvaccinated various feline species (three Sunda clouded leopard, two Leopard cats, 27 feral cats, and four Malayan tiger) were obtained from archives of Veterinary Laboratory Service Unit (VLSU), Faculty of Veterinary Medicine, Universiti Putra Malaysia. Since archive samples were used in this study, animal ethical approval was not required. The samples were received between the year 2014 and 2015 from zoos located in Sabah, Selangor, and Pahang for diagnostic purposes and general screening, and a year later, the archived sera were used in this study. Briefly, 100 μl of each felid serum was seeded in the 75% confluent Crandell-Rees feline kidney (CRFK) (ATCC CCL94) monolayer cell line in 25-cm^3^ cell culture flasks (Nunc, Germany). Then, the samples were incubated at 37°C with 1 ml of growth medium for 1 h at 5% CO_2_. After an hour of incubation, the growth medium was decanted and replaced with PBS to wash the cells thrice. Following that, 5 ml of MEM (GibcoBRL, USA) supplemented with 1% FBS (GibcoBRL, USA) (maintenance media) was added. The inoculated cells were then incubated again in 37°C incubators supplemented with 5% CO_2_ and were monitored daily for any appearance of cytopathic effect (CPE) in three blind passages. Any serum inoculated cells with CPE were further harvested and subjected to viral genome extraction.

### PCR and RT-PCR Analysis

Samples that showed consecutive CPE were subjected to viral DNA and RNA extraction using DNeasy Blood and Tissue Kit (Qiagen, Germany) and RNeasy Kit (Qiagen, Germany), respectively, according to the manufacturer's protocol. The extracted DNA was subjected to FHV and CPV PCR protocol, whereas, RNA was subjected to FCV and CDV RT-PCR protocol in a Mastercycler® gradient thermal cycler (Eppendorf, Germany). Information on the target genes, primer sequences used, positive control, PCR, and RT-PCR protocol, and expected amplicon size are shown in Supplementary Table 1 (15–18). Gel electrophoresis using 1.5% (w/v) agarose gel (Vivantis, USA) and 6× Loading Dye (Fermentas, USA) was conducted to view the amplification of positive reactions visualized by the presence of an amplicon fragment between target genes aligned with a positive control band using a UV transilluminator (Biorad, USA).

### Complete Genome Sequence of CPV

Viral DNA of CPV was extracted from the viral isolate, and the entire ORF (including NS1 and VP2 genes for CPV) was amplified using four pairs of primers (Supplementary Table 2). Primers for the virus genome sequence were designed using Primer-BLAST along with its specificity information (https://www.ncbi.nlm.nih.gov/tools/primer-blast/), according to the reference of a full complete sequence of a CPV isolate obtained through GenBank (NC_001539).

Positive isolate was run by PCR using designated primers targeted for each region of the virus genome. Primer target regions for PCRs for the different gene segments were selected from the conserved regions of the respective aligned gene sequences. Single intact band observed using agarose gel electrophoresis was sent for Sanger sequencing (Apical Scientific Sdn. Bhd, Malaysia).

### Phylogenetic Analysis

Eighty complete genome sequences of the viruses from the subfamily of *Parvovirinae* that represents eight different genera were included in this study (Supplementary Table 3). The sequences were aligned by using the online tool MAFFT version 7. The Neighbor-Joining tree was constructed with 1,000 replications using Mega-7 with Jukes-Cantor as substitution model. The Newick Export file was generated using MEGA-7, and the tree was viewed and edited using Interactive Tree of Life (iTOL) V5.

### Estimation of Divergence

About 176 and 181 of CP and NS genes from PPV, FPV, and CPV were retrieved from GenBank (Supplementary Table 4). The divergence times for parvoviruses were identified by the Bayesian Markov chain Monte Carlo (MCMC) method as applied in BEAST2 (Version 2.6.2) (19). The investigation was done under the GTR + I + G substitution model for C and NS gene sequence and used an unrelaxed log-normal-distributed (Ucld) relaxed molecular clock. The MCMC run was at 3 × 10^7^ steps long, with sampling at every 3,000 steps. Convergence was measured using Tracer software version 1.7.1 (available from http://beast.bio.ed.ac.uk/Tracer) by examining the Effective Sample Sizes (ESS > 200), and the 95% HDP values provide the degree of uncertainty in each parameter estimate. To reconstruct plausible ancestral states, all nodes in the MCC tree were annotated with posterior probability values using TreeAnnotator v.2.6.2, and the tree was visualized with FigTree v1.4.4 (http://tree.bio.ed.ac.uk/software/figtree). The demographical history of CPV NS and capsid was estimated using the Bayesian skyline plot method applied in Tracer V1.7.1.

### Selection Pressure Analysis

The selection pressure of NS and CP genes from CPV was used to estimate the distinction between the non-silent site (dN) and silent site (dS) substitution rates by using MEGA version 7.0 software (20). The parameter was set as follows: dN/dS ratio >0 is positive selection; dN/dS ratio <0 is negative selection; and dN/dS ratio equal to 0 is neutral evolution (21). This ratio will measure the selection pressures by equating the rate of substitutions at silent sites (dS), which are assumed neutral, to the rate of substitutions at non-silent sites (dN), which are probably involved in selection (22). The entropy was used to calculate the genetic density and determined by using BioEdit vision 7.2.5. The amino acid sequences of Protoparvovirus were compared with CPV capsid and NS genes.

## Results

### CPE and Virus Detection by PCR

**Figure 1:**
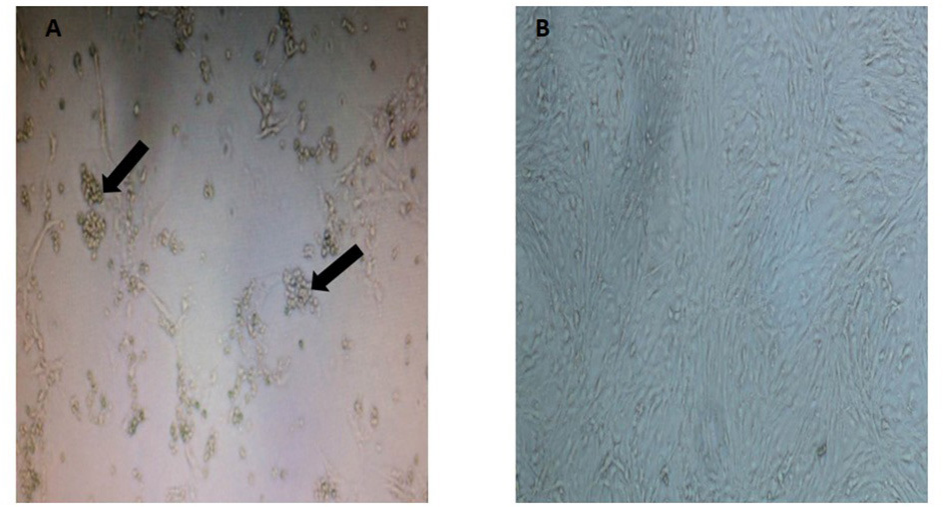
Appearance of cytopathic effect in CRFK cells in **(A)** a Malayan tiger (MT4), observed as clumping of cells with cluster formation and cytoplasmic extension (indicated by arrows at 8 d.p.i.) and **(B)** mock-infected control CRFK (magnification at 40×).

As observed in Figure 1, the CRFK cells from a Malayan tiger showed formation of CPE evidenced by cytoplasmic extension and clusters formation. Samples that tested positive with three consecutive CPEs were tested by PCR using virus-specific primers to detect FHV, FCV, CDV, and CPV. Only one sample of Malayan tiger species was positive for CPV. A single DNA band of the expected size (583 bp), corresponding to the partial amplification of VP2 gene of the CPV, was detected following gel electrophoresis.

### Full-Genome Sequencing of UPM-CPV15/P. Tigris Jacksoni

The positive isolate denoted as UPM-CPV15/P. tigris jacksoni was approximately 4,712 base pairs (bp) in size (GenBank Accession number MW380384). The complete genome sequence of isolate UPM-CPV15/P. tigris jacksoni was checked for specificity using BLAST (blast.ncbi.nlm.nih.gov/Blast.cgi). The specificity of isolate UPM-CPV15/P. tigris jacksoni was found to be highly similar to CPV-31 (GenBank accession no: M24000.1), CPV-d Cornell #320 (GenBank accession no: M23255.1), and CPV-15 (GenBank accession no: M24003.1) strains with 99.13, 98.65, and 98.40%, respectively, and classified as CPV-2a.

### Bioinformatic Analysis of UPM-CPV15/P. Tigris Jacksoni

**Figure 2:**
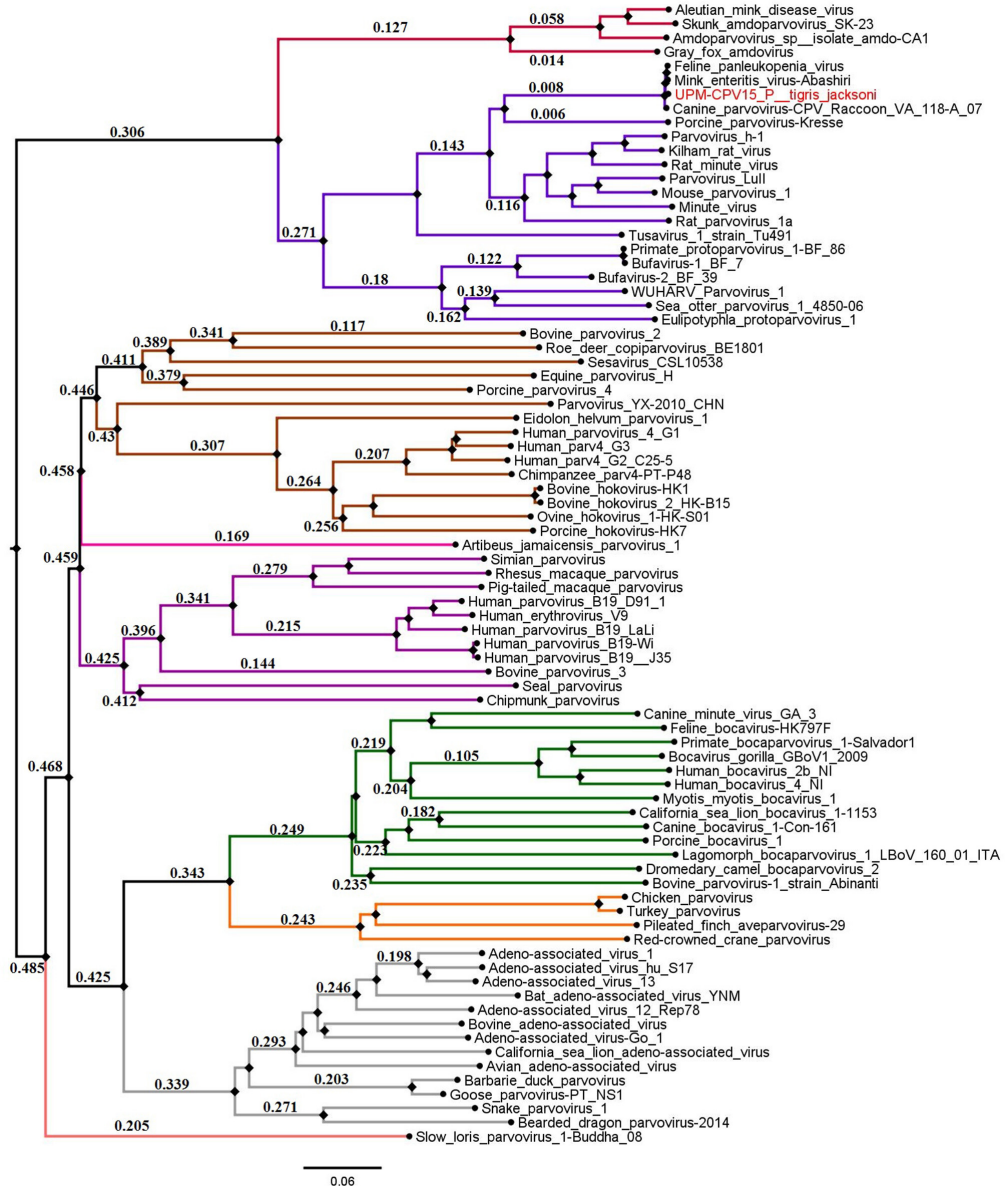
Neighbor joining tree of the subfamily *Parvovirinae*. The branches of the tree was colored according to the genera; *Bocaparvovirus* was colored forest green, *Amdoparvovirus* was colored carmine red, *Protoparvovirus* was colored electric violet, *Aveparvovirus* was colored orange, *Dependoparvovirus* was colored gray, unclassified was colored baby blue, *Erythroparvovirus* was colored Byzantium violet, and *Tetraparvovirus* was colored cinnamon brown. Local isolate strains UPM-CPV15/P. tigris jacksoni were highlighted in red.

The phylogenetic tree was constructed for viruses from different genera of the subfamily *Parvovirinae*, including *Amdoparvovirus, Aveparvoviru*s, *Bocaparvoviru*s, *Copiparvovirus, Dependoparvovirus, Erythroparvovirus, Protoparvovirus*, and *Tetraparvovirus*, which were used to create a Neighbor-Joining phylogenetic tree. The complete genome of UPM-CPV15/P. tigris jacksoni shares the same branch with CPV, FPV, and MEV under the genus *Protoparvovirus* (Figure 2).

**Figure 3:**
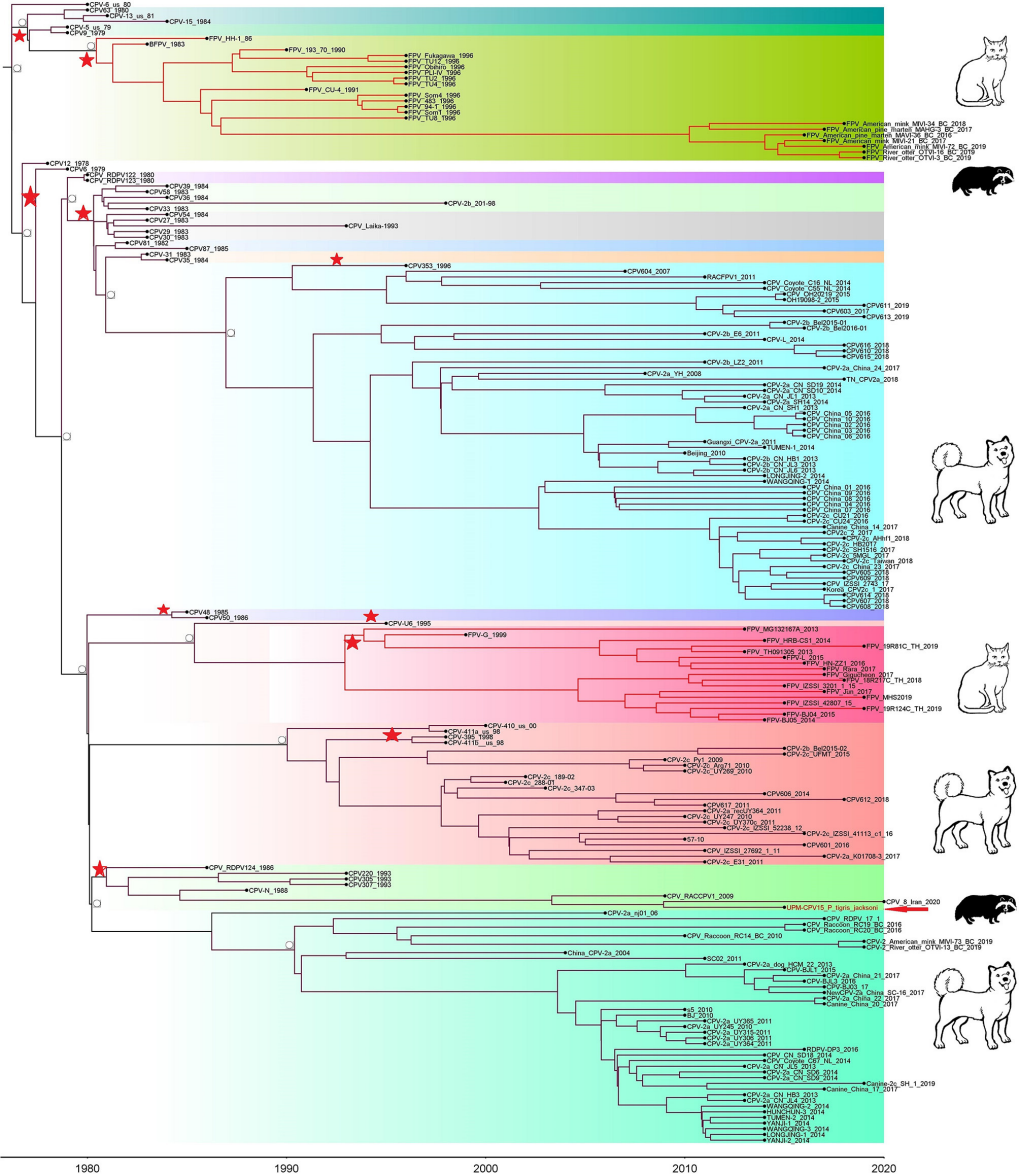
Time-scaled Bayesian Maximum Clade Credibility tree for the non-structure (NS) genes of porcine, canine, and feline panleukopenia viruses. The local isolate was highlighted in golden color. PPV, CPV, and FPV were highlighted in red, green, and blue color, respectively. Each branch was colored according to posterior probability (PP) value; blue bars at nodes indicate 95% highest probability density (HPD). Tree nodes with a posterior probability >0.8 was displayed at tree nodes with red color.

**Figure 4:**
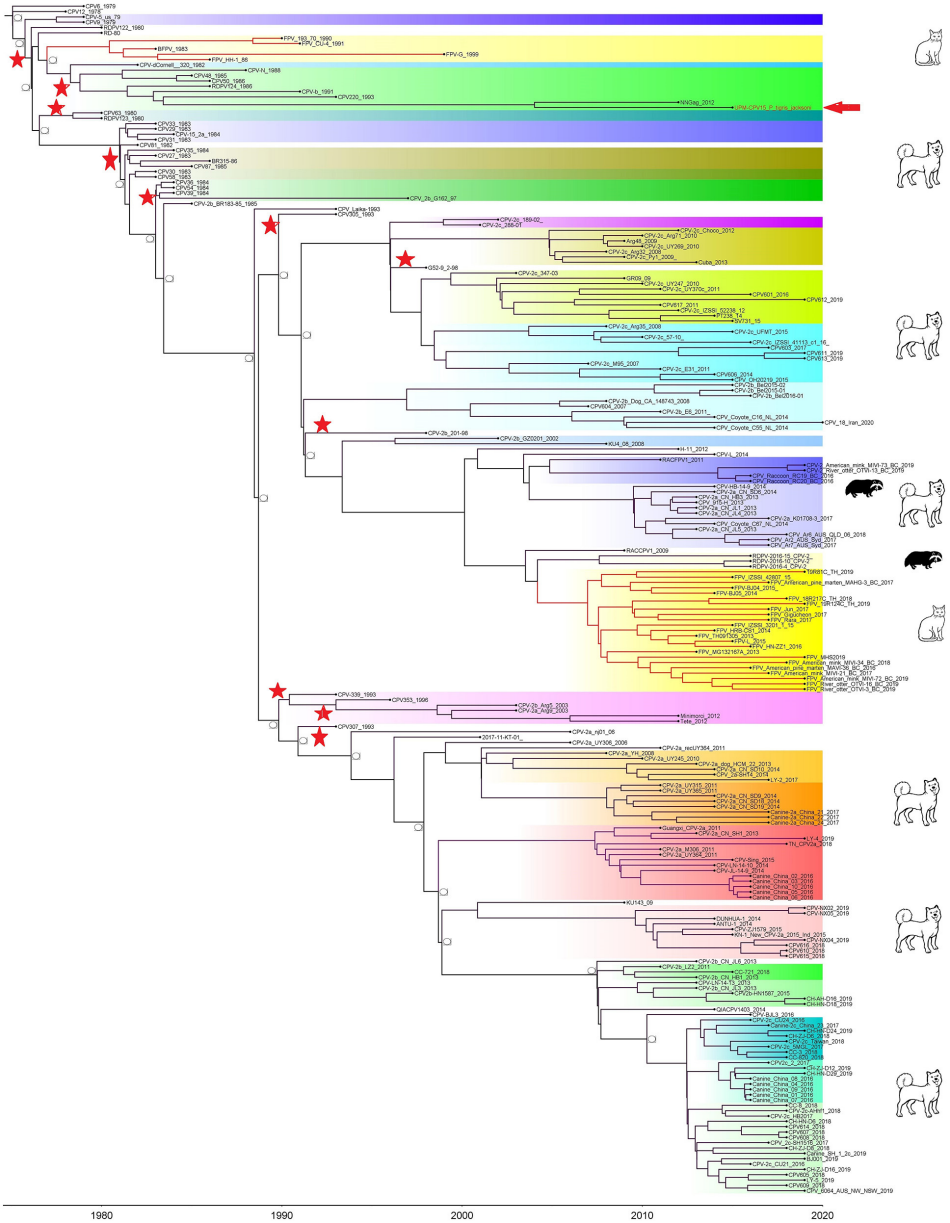
Time-scaled Bayesian Maximum Clade Credibility tree for the capsid genes (VP) of porcine, canine, and feline panleukopenia viruses. The local isolate was highlighted in golden color. PPV, CPV, and FPV were highlighted in red, green, and blue color, respectively. Each branch was colored according to posterior probability (PP) value; blue bars at nodes indicate 95% highest probability density (HPD). Tree nodes with a posterior probability >0.8 was displayed at tree nodes with red color.

Phylogenetic trees were constructed for CPV capsid protein CP (VP 1 and VP2) and NS gene using 223 and 201 sequences, respectively, from the 1970's to 2020, from countries in North and South America, Asia, and Europe (Figures 3, 4). The tree was scaled to time (years); the time to the most recent common ancestor (tMRCA) with its corresponding 95% highest posterior density (HPD) interval for CPV was estimated in the unit of years. The tMRCA tress of 11 and 25 major branches for NS and CP genes, respectively, as highlighted in different colors (Figures 3, 4). The NS gene of UPM-CPV15/P. tigris jacksoni shared the same branch with CPV8 Iran strain isolated in 2020 from Iran. Both UPM-CPV15/P. tigris jacksoni and CPV_8_Iran strains shared the same node with raccoon-related CPV, which is isolated in 2009 (Figure 3), while the tMRCA of NS and CP genes of CPV and FPV was predicted to originate in the late 1970's (95% HPD: 1973–1982) and (95% HPD: 1973–1979), respectively. The CP gene of UPM-CPV15/P. tigris jacksoni shared the same branch with NNGag isolated in 2012 from Argentina (Figure 4). Both NNGag and UPM-CPV15/P. tigris jacksoni shared the same node with CPV220, which was detected from dog in 1993 from USA. This clade consisted of the older clade (red star) of CPV before the year 2000.

**Figure 5:**
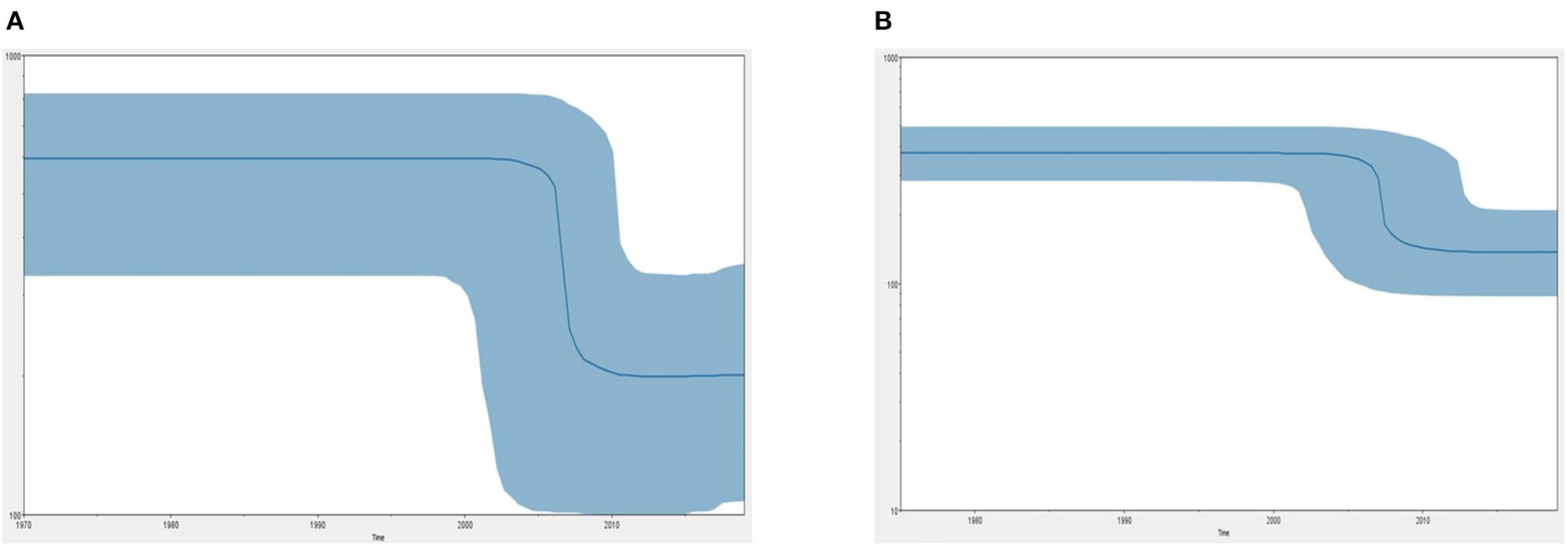
Bayesian skyline plot (BSP) shows the changes in effective population size over time of canine parvovirus. **(A)** The demographical history of the canine parvovirus NS gene. **(B)** The demographical history of the canine parvovirus capsid gene. The blue line denotes the median posterior value, and the blue area represents the 95% HPD intervals. The *x*-axis is labeled years (time) and the *y*-axis is labeled effective population size.

Figure 5 demonstrates the demographic history of CPV NS and CP genes. An S-shape was observed over the 41 years (1978 to 2019). Both NS (a) and capsid (b) genes of CPV populations have had a stable size until the first decline inferred range between the years 2000 and 2010. A steady population size followed the decrease until the current date. Table 1 shows the evolution rate of the CP (VP 1 and VP2) and NS gene of *Protoparvovirus*. Overall, the capsid of PPV, FPV, and MPV shows higher nucleotide substitutions/site/year. While the NS gene showed lower nucleotide substitutions/site/year as compared to PPV, RPV, and FPV.

**Table 1:** Nucleotide substitution rates of capsid and NS gene of protoparvoviruses.

**Dataset**	**Nucleotide substitution rate**			
	**Capsid**	**95% HPD**	**NS**	**95% HPD**
CPV	1.649 × 10^−5^	7.626 × 10^−3^ to 7.440 × 10^−3^	1.203 × 10^−4^	6.663 × 10^−3^ to 6.593 × 10^−3^
PPV	8.154 × 10^−4^	1.377 × 10^−4^ to 1.375 × 10^−4^	1.027 × 10^−3^	1.065 × 10^−4^ to 1.065 × 10^−4^
FPV	1.381 × 10^−4^	7.4115 × 10^−3^ to 7.325 × 10^−3^	1.1 × 10^−4^	6.858 × 10^−3^ to 6.858 × 10^−3^
MEV	8.884 × 10^−5^	3.675 × 10^−3^ to 3.613 × 10^−3^	1.71 × 10^−5^	3.196 × 10^−3^ to 3.125 × 10^−3^
MPV	1.263 × 10^−4^	7.653 × 10^−3^ to 7.603 × 10^−3^	5.201 × 10^−5^	7.133 × 10^−3^ to 7.074 × 10^−3^
RPV	5.794 × 10^−5^	1.067 × 10^−4^ to 1.062 × 10^−4^	2.309 × 10^−4^	9.549 × 10^−3^ to 9.509 × 10^−3^

**Figure 6:**
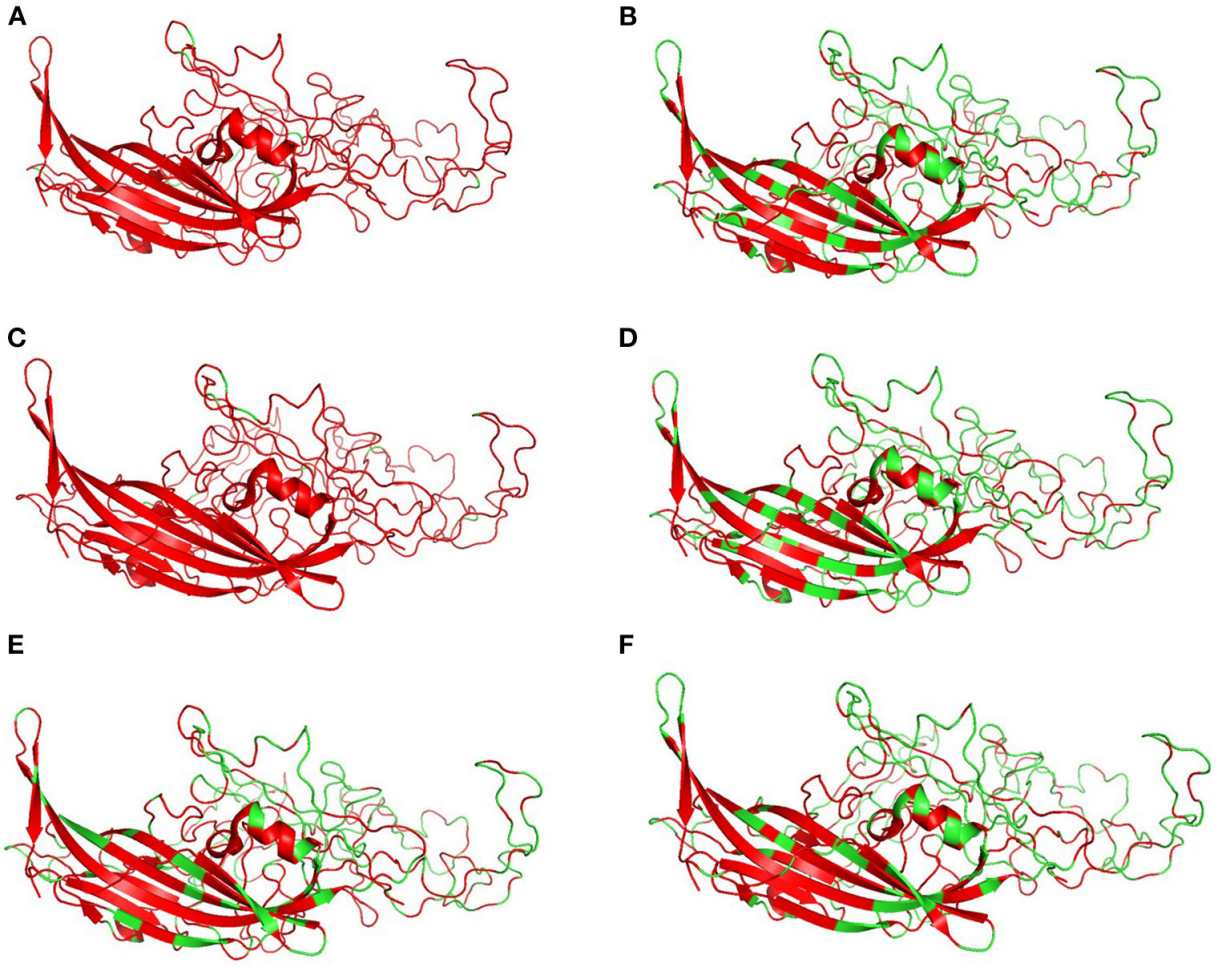
Changes in capsid: Three-dimensional structure of canine parvovirus capsid comparison with other *Protoparvovirus*. Red indicates the portion of similar amino acids shared, whereas, green is dissimilar amino acids. Comparison of CPV capsid protein with **(A)** FPV, **(B)** KRV, **(C)** MEV, **(D)** MPV, **(E)** PPV, and **(F)** RPV.

**Figure 7:**
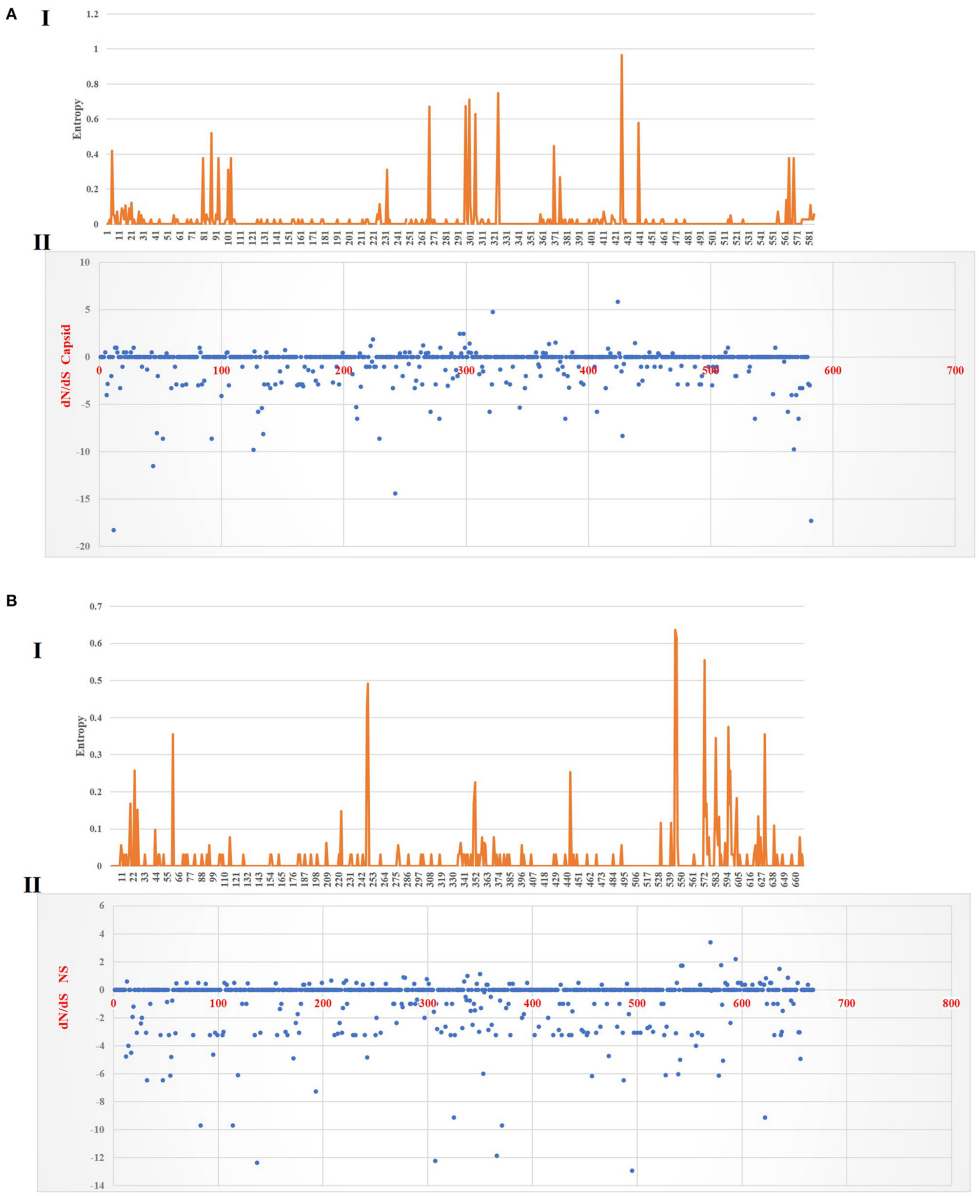
Plot for the distinction between non-synonymous and synonymous rates (dN–dS) and amino acid entropy rate for the CP and NS genes. The entropy for CP amino acid sequences shows exhibited high-level entropy [**(A)** I] and the dN–dS ratio falls under purified selection [**(A)** II]. The entropy for NS amino acid sequences shows exhibited low-level entropy [**(B)** I] and the dN–dS ratio falls under purified selection [**(B)** II].

Figure 6 shows the three-dimensional structure comparison of CPV capsid and NS gene with other *Protoparvovirus*. The dN/dS ratio in this study showed that most codons fell under neutral or negative selection for both CP and NS genes (Figures 7A,B). High levels of variability were spotted at CP amino acid sequences compared to NS. The CP protein amino acid sequence was at the 4, 80–87, 93–103, 232, 267, 297, 300–305, 323–324, 370–375, 426, 440, and 564–568 position. Comparing the amino acid sequence of CPV capsid and NS genes with other *Protoparvovirus* shows that only FPV and MEV show high similarity, whereas, PPV, MPV, KRV, and RPV show less similarity with CPV (Table 2).

**Table 2:** Percentage of amino acid similarity with CPV non-structural and capsid proteins.

**Protoparvoviruses**	**Percentage of amino acid similarity with CPV (%)**	
	**NS**	**Capsid**
FPV	98.5	98
MEV	98.2	98
PPV	66.9	59.2
MPV	72.6	53
KRV	73.5	52.9
RPV	79.7	54.3

## Discussion

Wild felids are susceptible to virus infection due to cross-species transmission potentially by domestic cats or dogs (23). Early detection of viral shedding of asymptomatic or symptomatic felids may help in elucidating the health status of wild felids in captive or nature. Since the status of virus infections among captivated wild felids in Malaysia is lacking, we aimed in this study to screen the presence of FHV, FCV, CDV, and CPV from serum samples in the leopard, feral cat, and tiger from zoo. Isolation based on cell cultures and detection of the viruses by gene-specific PCR/RT-PCR and full genome sequence analysis of the CPV isolate were carried out.

In general, parvovirus, especially CPV, has higher tropism in rapidly dividing cells such as the crypt of lieberkuhn in the intestine and is associated with clinical manifestation of diarrhea and gastroenteritis (1). In this study, serum sample was used instead of fecal sample primarily because the study was aimed to screen the presence of virus shedding from healthy captive felids. Several studies have included serum or plasma other than fecal or intestine tissue to diagnose presence of parvovirus DNA in human (24, 25), dog (26), and feline (27). Elucidating the status of virus shedding in the captive wild felids is part of a surveillance program that essentially will help in preventing occurrence of infectious disease outbreaks in the captivated wildlife.

Isolation of virus from various sample sources from either human, animal, or environment based on cell culture technique has been used extensively. However, in most cases, further, confirmatory analysis based on molecular tests is required to identify the virus since the detection of CPEs is a general indicator of viral-induced cellular changes and often offers inconclusive diagnosis (28). Isolation in CRFK cells was chosen as it is the most commonly used cell line for virus isolation in mostly feline samples, which include feline foamy virus (29), feline picornaviruses (30), FPV (31), feline leukemia virus (FeLV) (32), FHV (33), and CPV. Of the 36 serums inoculated in CRFK, 11 samples showed positive CPE up to three consecutive passages, whereas, only one sample was positive for PCR detection based on virus-specific primer combinations that detect CPV from a Malayan tiger. The other 10 isolates that were negative for PCR detection in the tested viruses were probably associated with unknown viruses or other types of viruses that were not included in this study, such as the feline immunodeficiency virus (FIV), FeLV, or feline coronavirus (FCoV).

In this study, UPM-CPV15/P. tigris jacksoni isolate was obtained through direct sequencing protocol based on Sanger sequencing. This isolate's complete genome was highly similar to CPV-31, CPV-d Cornell #320, and CPV-15 with 99.13, 98.65, and 98.40%, respectively. These strains are classified as CPV-2a and originated from USA. CPV-2a, however, is a very common CPV variant isolated from Asia (34). Although, the current strain was isolated in 2015, CPV-31, CPV-d Cornell #320, and CPV-15 were isolated in the early 1980's. Nevertheless, the CPV-31 strain shows high similarity with strains isolated from Beijing and Italy, namely, CPV-L (99.78%) (GenBank accession: MG763189.1) in 2014 and CPV_IZSSI_2323_11 (99.56%) (GenBank accession: KX434458.1) in 2011, indicating that the virus is still circulating, although, those strains were discovered three decades ago. Meanwhile, Wang et al. (35) and Jiao et al. (36) isolated parvovirus from the captive Siberian tigers, which was closely related to racoon parvovirus, indicating the susceptibility of felids to parvovirus. In regard to variant, CPV-2a and CPV-2b have been isolated from cheetah and tiger while CPV-2c was isolated from leopard (37, 38).

The Neighbor-Joining method is a suitable model to study evolution and has a well-chosen maximum likelihood model parameter that can often produce a reasonably good phylogenetic tree (39). Because of that, the Neighbor-Joining analysis method was chosen in order to better understand how divergent was isolate from this study within the species in the same genera and to verify that it clustered together with CPV. In genome diversity, mutation rate is an important parameter, which is the per-nucleotide or amino acid site mutation rate and the genome size. This mutation rate will determine the average number of mutations each offspring will have compared to the parental (or ancestral) genome. In this study, the phylogeny of the full length of the CP and NS gene was constructed separately based on the availability of sequences in GenBank. Comparing both phylogenetic tree, CP shows more branches as compared to NS. Variation in the CPV capsid from this study was also observable with FPV, a common variant of CPV. The structural protein CP (VP2 protein) produces multiple mutations during the evolution, causing more amino acid variation; thus, CP is denoted as the main antigenic determinant of CPV as it plays a key role in the mutation and changes in the capsid's amino acid sequences for adaptation to the host cellular receptors (40). Similarly, this study demonstrated higher nucleotide and amino acid sequence mutation in CP sequences as compared to the NS gene when compared with other species in *Protoparvoviruses* (Tables 1, 2).

Multiple statistical tests have been established to measure selection pressures that play a role in protein-coding regions. The dN/dS ratio is commonly used, owing in part to its simplicity and robustness. The dN/dS ratio in this study showed that most codons fell under neutral or less positive selection for both CP and NS genes. These findings are expected since positive selection and genetic recombination occur frequently in the parvovirus (41–43). High levels of variability were spotted at CP amino acid sequences as compared to NS. The CP protein amino acid sequence at position 4, 80–87, 93–103, 232, 267, 297, 300–305, 323–324, 370–375, 426, 440, and 564–568 demonstrated high complexity. This is comparable with the value based on the previous study by Zhou et al. (34), which is expecting 10^−4^ substitution of nucleotides per site per year, and amino acid mutation at position 267, 324, and 440. More variations observed in the phylogeny and amino acid sequences of the capsid of CPV strain from this study further articulate its crucial role in interacting with specific host cellular receptor for successive entry and penetration during replication. Mutation could be the main influence that changes the capsid's amino acid sequences for adaptation to the host cellular receptors (41). Meanwhile, the high similarity of current CPV NS amino acid sequences and phylogeny might be due to their definite role in the DNA replication mechanism (44).

Phylo-dynamics of CPV is important in an effort to shed light on how the transmission dynamics impact viral genetic differences and deliver important insights into epidemiology and virus evolution. To discover the demographic history of the CPV in the tested population over the time period, the relative actual population size over time was inferred by investigating the genetic diversity using the Bayesian skyline model. The demographic pattern of CPV NS and capsid dropped after the 2000's to 2010's (Figure 5). This could be explained by the reduction in the infection rate of the infected population due to immunization of inactivated and live vaccines that are used widely in dogs (12). However, vaccination against CPV could be responsible for the occurrence of antigenic variants through antigenic drift events to evade the host immune system (41, 45–49). Postulating exactly which animals specifically are the susceptible hosts for CPV and FPV particularly and how the genetic composition of the viruses will change in the different hosts are still unclear. Nevertheless, diverse extension of pathways and emergence of novel strain(s) might be contributed by several dynamics such as animal movement and the presence of the carrier or intermediate host that spread the virus causing cross-species transmission at large spatial scales worldwide (50). Thus far, a relatively low mutation has been observed in CPV over 40 years even though, rapid host adaptation was common, suggesting that continuous high diversities of genetic sequences of host between same species or interspecies are not necessarily required for virus host adaptation in CPV (51).

## Conclusion

In conclusion, this study reports the presence of CPV (CPV-2a) in a Malayan tiger. Full-genome analysis denotes that the present isolate size is ~4.7 kbp and encodes for two major genes, the NS and the capsid gene. Genome sequencing provides valuable information on the genomic characteristics of the CPV from this study and other viruses from the *Protoparvovirus* genus.

## Data Availability Statement

The original contributions presented in the study are included in the article/Supplementary Material, further inquiries can be directed to the corresponding author/s.

## Ethics Statement

Ethical review and approval was not required for the animal study because the samples used were archived and received for diagnostic screening.

## Author Contributions

AN-F and KK were involved in writing the original draft preparation and conducted methodology by using software. AY, AO, and SC were involved in supervision, conceptualization, and writing in terms of reviewing and editing. All the authors have read and approved the final manuscript.

## Conflict of Interest

The authors declare that the research was conducted in the absence of any commercial or financial relationships that could be construed as a potential conflict of interest.

## Publisher's Note

All claims expressed in this article are solely those of the authors and do not necessarily represent those of their affiliated organizations, or those of the publisher, the editors and the reviewers. Any product that may be evaluated in this article, or claim that may be made by its manufacturer, is not guaranteed or endorsed by the publisher.
