# Supplementary material for: Isolation and Genetic Characterization of Canine Parvovirus in a Malayan Tiger
Source: Front Vet Sci. 2021 Aug 3;8:660046. doi: 10.3389/fvets.2021.660046 (PMC8369201; doi:10.3389/fvets.2021.660046)
Supplement: Supplementary file 1 [file Data_Sheet_1.docx]

**Supplementary table 1**: List of viruses targeted in this study and their target gene and primer sequence

| **Virus** | **Target gene** | **Primer Sequence** | **Amplified fragment** | **References** |
| --- | --- | --- | --- | --- |
| Feline Herpesvirus (FHV) | TK F  TK R | **F**-GACGTGGTGAATTATCAGC  **R**-CAACTAGATTTCCACCAGGA | 292 bp | 14 |
| Feline Calicivirus  (FCV) | Cali1  Cali2 | **F**-AACCTGCGCTAACGTGCTTA  **R**-CAGTGACAATACACCCAGAAG | 924 bp | 15 |
| Canine Distemper  virus (CDV) | Nfp  Nrp | **F**-GCTGGTTGGAGAATAAGG  **R**-CCAACTCCCATAGCATAA | 586 bp | 16 |
| Canine Parvovirus  (CPV) | VP2  VP2 | **F**-CAGGAAGATATCCAGAAGGA  **R**-GGTGCTAGTTGATATGTAATAAACA | 583 bp | 17 |

**Supplementary table 2**: Primers used for genome sequencing of canine parvovirus for positive isolate

| **Primers** | **Primer sequence (5’-3’)** | **Nucleotide positions** | **Expected size (bp)** |
| --- | --- | --- | --- |
| CPV 3a F | ACACGTCATACGTACGCTCC | 73-92 | 2500 |
| CPV 3a R | GACCACCGTCTGGTTGAACT | 2814-2995 |  |
| CPV 3b F | AACTAACCCTTCTGACGCCG | 2442-2461 | 2500 |
| CPV 3b R | CAAACCAACCAACCACCCAC | 4762-4743 |  |
| CPV 3’ F | ATGATGGCACAACCAGGAGG | 1211-1230 | 1600 |
| CPV 3’ R | ACCCGTTCCCAGATCCTGTA | 2864-2845 |  |
| CPV 3’’ F | AGAGCATTGGGCTTACCACC | 3632-3651 | 1300 |
| CPV 3’’ R | CAACCAACCACCCACACCAT | 4949-4930 |  |

**Supplementary Table 3**: Details of the sub-family of *Parvovirinae* used for the phylogenetic analysis

| **No** | **Acession no** | **virus** |
| --- | --- | --- |
| 1 | JN040434 | Aleutian mink disease virus |
| 2 | GU214704 | Chicken parvovirus |
| 3 | GU214706 | Turkey parvovirus |
| 4 | FJ214110 | Canine minute virus |
| 5 | JN420361 | California sea lion bocavirus 1 |
| 6 | JQ923422 | Primate bocaparvovirus 1 |
| 7 | HM145750 | Bocavirus gorilla |
| 8 | FJ973560 | Human bocavirus 2b |
| 9 | FJ973561 | Human bocavirus 4 NI |
| 10 | DQ335247 | Bovine parvovirus-1 |
| 11 | AF406966 | Bovine parvovirus 2 |
| 12 | AF063497 | Adeno-associated virus 1 |
| 13 | AY388617 | Bovine adeno-associated virus |
| 14 | DQ335246 | Adeno-associated virus-Go.1 |
| 15 | JF926695 | Goose parvovirus-PT |
| 16 | AY186198 | Avian adeno-associated virus |
| 17 | GU226971 | Bat adeno-associated |
| 18 | JN420372 | California sea lion adeno-associated virus |
| 19 | AY349010 | Snake parvovirus 1 |
| 20 | U26342 | Simian parvovirus |
| 21 | AF221122 | Rhesus macaque parvovirus |
| 22 | AF221123 | Pig-tailed macaque parvovirus |
| 23 | AF406967 | Bovine parvovirus 3 |
| 24 | JQ037753 | Eidolon helvum parvovirus 1 |
| 25 | AY622943 | Human parvovirus 4 G1 |
| 26 | HQ113143 | Chimpanzee parv4 |
| 27 | EU200669 | Bovine hokovirus |
| 28 | EU200677 | Porcine hokovirus |
| 29 | JF504699 | Ovine hokovirus 1 |
| 30 | EU659111.1 | Feline panleukopenia virus |
| 31 | D00765.1 | Mink enteritis virus |
| 32 | JN867610.1 | racoon parvovirus |
| 33 | JX027296.1 | Primate protoparvovirus 1 |
| 34 | JX027295.1 | Bufavirus-1 |
| 35 | JX027297.1 | Bufavirus-2 |
| 36 | X01457.1 | Parvovirus h-1 |
| 37 | AF321230.1 | Kilham rat virus |
| 38 | M81888 | Parvovirus LuII |
| 39 | J02275.1 | Minute virus |
| 40 | U12469 | Mouse parvovirus |
| 41 | AF332882.1 | Rat minute virus |
| 42 | AF036710 | rat parvovirus 1 |
| 43 | U44978 | porcine parvovirus Kresse |
| 44 | GU938300 | porcine Cn virus |
| 45 | JF504697 | Bovine hokovirus 2 |
| 46 | EU874248 | human parv4 G3 |
| 47 | DQ873391 | Human parv4 G2 |
| 48 | AY083234 | human parvovirus B19-D91 |
| 49 | AJ249437 | Human erythrovirus V9 |
| 50 | AY044266 | Human parvovirus B19 |
| 51 | M24682 | Human parvovirus B19-Wi |
| 52 | AY386330 | human parvovirus B19-J35 |
| 53 | AY695376 | adeno-associated virus-S17 |
| 54 | EU285562 | Adeno-associated virus 13 |
| 55 | DQ813647 | Adeno-associated virus 12 |
| 56 | KP120516.1 | Slow loris parvovirus 1 |
| 57 | JQ037754.1 | Artibeus jamaicensis parvovirus 1 |
| 58 | KY312546 | Red-crowned crane parvovirus |
| 59 | KY640438 | Dromedary camel bocaparvovirus 2 |
| 60 | KU561552.1 | Sea otter parvovirus 1 |
| 61 | MK091524.1 | Roe deer copiparvovirus |
| 62 | MG136722.1 | Equine parvovirus H |
| 63 | KM035804.1 | Sesavirus |
| 64 | KF373759.1 | Seal parvovirus |
| 65 | KT878839.1 | red panda Amdoparvovirus |
| 66 | KJ495710.1 | Tusavirus 1 |
| 67 | JN648103.1 | Canine bocavirus 1 |
| 68 | JQ692585.1 | Feline bocavirus |
| 69 | HM053693.2 | Porcine bocavirus 1 |
| 70 | KP729195.1 | Lagomorph bocaparvovirus 1 |
| 71 | JQ814850.1 | Myotis myotis bocavirus 1 |
| 72 | MG745672.1 | Pileated finch aveparvovirus |
| 73 | AB937988.1 | Eulipotyphla protoparvovirus |
| 74 | JX627576.1 | WUHARV Parvovirus 1 |
| 75 | KX981923.1 | Skunk amdoparvovirus |
| 76 | JN202450.1 | Gray fox amdovirus |
| 77 | U22967.1 | Barbarie duck parvovirus |
| 78 | KP733794.1 | Bearded dragon parvovirus |
| 79 | GQ200736.1 | Chipmunk parvovirus |

**Supplementary Table 4**: Details of the canine and feline panleukopenia viruses strains used for the time-scaled Bayesian Maximum Clade Credibility tree

| **No** | **Accession no** | **Strain** | **Year** | **host** | **Note** | **Type** |
| --- | --- | --- | --- | --- | --- | --- |
| 1 | MG013488.1 | CPV-SH1516 | 2017 | dog | complete | 2c |
| 2 | KT382542.1 | CPV-SH14 | 2014 | dog | complete | 2a |
| 3 | MN840830.1 | Canine/SH/1/2019 | 2019 | dog | complete | 2c |
| 4 | MK388674.1 | HB2017 | 2017 | dog | complete | 2c |
| 5 | MN451695.1 | RDPV124 | 1986 | Raccoon dog | complete | - |
| 6 | MN451694.1 | RDPV123 | 1980 | Raccoon dog | complete | - |
| 7 | MN451693.1 | RDPV122 | 1980 | Raccoon dog | complete | - |
| 8 | MN451691.1 | RACCPV1 | 2009 | Raccoon | complete | - |
| 9 | MN451681.1 | CPV608 | 2018 | dog | complete | - |
| 10 | MN451680.1 | CPV607 | 2018 | dog | complete | - |
| 11 | MN451685.1 | CPV612 | 2019 | dog | complete | - |
| 12 | MN451687.1 | CPV614 | 2018 | dog | complete | - |
| 13 | MN451688.1 | CPV615 | 2018 | dog | complete | - |
| 14 | MN451689.1 | CPV616 | 2018 | dog | complete | - |
| 15 | MN451690.1 | CPV617 | 2011 | dog | complete | - |
| 16 | MN451674.1 | CPV353 | 1996 | dog | complete | - |
| 17 | MN451673.1 | CPV307 | 1993 | dog | complete | - |
| 18 | MN451669.1 | CPV81 | 1982 | dog | complete | - |
| 19 | MN451666.1 | CPV54 | 1984 | dog | complete | - |
| 20 | MN451663.1 | CPV39 | 1984 | dog | complete | - |
| 21 | MN451660.1 | CPV33 | 1983 | dog | complete | - |
| 22 | NC_001539.1 | CPV-N | 1988 | dog | complete | - |
| 23 | KY403998.1 | CPV-YH | 2008 | dog | complete | 2a |
| 24 | MF423125.1 | CPV/Coyote/C67/NL_2014 | 2014 | Coyote | complete | - |
| 25 | MF423124.1 | CPV/Coyote/C55/NL_2014 | 2014 | Coyote | complete | - |
| 26 | MF423123.1 | CPV/Coyote/C16/NL_2014 | 2014 | Coyote | complete | - |
| 27 | JN033694.1 | Laika-1993 | 1993 | dog | complete | - |
| 28 | MN862742.1 | CPV-2/River otter/OTVI-13/BC_2019 | 2019 | North American river otter | complete | - |
| 29 | MN862741.1 | CPV-2/American mink/MIVI-73/BC_2019 | 2019 | American mink | complete | - |
| 30 | M38245.1 | CPV-b | 1991 | dog | complete | - |
| 31 | MN908257.1 | MHS2019 | 2019 | South China tiger | complete | - |
| 32 | KX685354.1 | HN-ZZ1 | 2016 | tiger | complete | - |
| 33 | KX900570.1 | HH-1/86 | 1986 | jaguar | complete | - |
| 34 | KP769859.1 | MG132167A | 2013 | cat | complete | - |
| 35 | KP280068.1 | HRB-CS1 | 2014 | cat | complete | - |
| 36 | M38246.1 | CU-4 | 1991 | cat | complete | - |
| 37 | AB000067.1 | TU4 | 1996 | cat | complete | - |
| 38 | AB000065.1 | TU2 | 1996 | cat | No Capsid | - |
| 39 | AB000063.1 | TU12 | 1996 | cat | No Capsid | - |
| 40 | AB000060.1 | Som4 | 1996 | cat | No Capsid | - |
| 41 | AB000058.1 | Som1 | 1996 | cat | No Capsid | - |
| 42 | AB000057.1 | PLI-IV | 1996 | cat | No Capsid | - |
| 43 | AB000055.1 | Obihiro | 1996 | cat | No Capsid | - |
| 44 | AB000053.1 | Fukagawa | 1996 | cat | No Capsid | - |
| 45 | AB000049.1 | 94-1 | 1996 | cat | No Capsid | - |
| 46 | AB000048.1 | 483 | 1996 | cat | No Capsid | - |
| 47 | X55115.1 | 193/70 | 1970 | cat | complete | - |
| 48 | MN400980.1 | Rara | 2017 | feline | complete | - |
| 49 | MN400979.1 | Jun | 2017 | feline | complete | - |
| 50 | MN400978.1 | Gigucheon | 2017 | feline | complete | - |
| 51 | MN862749.1 | FPV/River otter/OTVI-3/BC_2019 | 2019 | North American river otter | complete | - |
| 52 | MN862748.1 | FPV/River otter/OTVI-16/BC_2019 | 2019 | North American river otter | complete | - |
| 53 | MN862747.1 | FPV/American mink/MIVI-72/BC_2019 | 2019 | American mink | complete | - |
| 54 | MN862746.1 | FPV/American mink/MIVI-21/BC_2017 | 2017 | American mink | complete | - |
| 55 | MN862745.1 | FPV/American pine marten/MAHG-3/BC_2017 | 2017 | American marten | complete | - |
| 56 | MN862744.1 | FPV/American pine marten/MAVI-36/BC_2016 | 2016 | American marten | complete | - |
| 57 | MN862743.1 | FPV/American mink/MIVI-34/BC_2018 | 2018 | american mink | complete | - |
| 58 | MN127781.1 | 19R124C/TH/2019 | 2019 | cat | complete | - |
| 59 | MN127780.1 | 19R81C/TH/2019 | 2019 | cat | complete | - |
| 60 | MN127779.1 | 18R217C/TH/2018 | 2018 | cat | complete | - |
| 61 | MN451692.1 | RACFPV1 | 2011 | Raccoon | complete | - |
| 62 | MN451652.1 | BFPV | 1983 | Arctic fox | complete | - |
| 63 | KP019621.2 | TH091305 | 2013 | small Indian civet | complete | - |
| 64 | KX434461.1 | FPV_IZSSI_3201_1_15 | 2015 | cat | complete | - |
| 65 | KX434462.1 | FPV_IZSSI_42807_15 | 2015 | cat | complete | - |
| 66 | MH165482.1 | FPV-BJ05 | 2014 | cat | complete | - |
| 67 | MH165481.1 | FPV-BJ04 | 2015 | cat | complete | - |
| 68 | MG764511.1 | FPV-L | 2015 | Lion | complete | - |
| 69 | MG764510.1 | FPV-G | 1999 | tiger | complete | - |
| 70 | AB000069.1 | TU8 | 1996 | cat | No Capsid | - |
| 71 | KR002805.1 | CPV/CN/SD19/2014 | 2014 | dog | complete | 2a |
| 72 | KR002804.1 | CPV/CN/SD18/2014 | 2014 | dog | complete | 2a |
| 73 | KR002803.1 | CPV/CN/SD10/2014 | 2014 | dog | complete | 2a |
| 74 | KR002802.1 | CPV/CN/SD9/2014 | 2014 | dog | complete | 2a |
| 75 | KR002801.1 | CPV/CN/SD6/2014 | 2014 | dog | complete | 2a |
| 76 | KR002799.1 | CPV/CN/JL6/2013 | 2013 | dog | complete | 2b |
| 77 | KR002798.1 | CPV/CN/JL5/2013 | 2013 | dog | complete | 2a |
| 78 | KR002797.1 | CPV/CN/JL4/2013 | 2013 | dog | complete | 2a |
| 79 | KR002796.1 | CPV/CN/JL3/2013 | 2013 | dog | complete | 2b |
| 80 | KR002795.1 | CPV/CN/JL1/2013 | 2013 | dog | complete | 2a |
| 81 | KR002794.1 | CPV/CN/HB3/2013 | 2013 | dog | complete | 2a |
| 82 | KR002793.1 | CPV/CN/HB1/2013 | 2013 | dog | complete | 2b |
| 83 | KR002792.1 | CPV/CN/SH1/2013 | 2013 | dog | complete | 2a |
| 84 | MT010564.1 | CPV-AHhf1 | 2018 | dog | complete | 2c |
| 85 | MN832850.1 | Taiwan/2018 | 2018 | pangolin | complete | 2c |
| 86 | MN451686.1 | CPV613 | 2019 | dog | complete | - |
| 87 | MN451684.1 | CPV611 | 2019 | dog | complete | - |
| 88 | MN451683.1 | CPV610 | 2018 | dog | complete | - |
| 89 | MN451682.1 | CPV609 | 2018 | dog | complete | - |
| 90 | MN451679.1 | CPV606 | 2014 | dog | complete | - |
| 91 | MN451678.1 | CPV605 | 2018 | dog | complete | - |
| 92 | MN451677.1 | CPV604 | 2007 | dog | complete | - |
| 93 | MN451676.1 | CPV603 | 2017 | dog | complete | - |
| 94 | MN451675.1 | CPV601 | 2016 | dog | complete | - |
| 95 | MN451672.1 | CPV305 | 1993 | dog | complete | - |
| 96 | MN451671.1 | CPV220 | 1993 | dog | complete | - |
| 97 | MN451670.1 | CPV87 | 1985 | dog | complete | - |
| 98 | MN451668.1 | CPV63 | 1980 | dog | complete | - |
| 99 | MN451667.1 | CPV58 | 1983 | dog | complete | - |
| 100 | MN451665.1 | CPV50 | 1986 | dog | complete | - |
| 101 | MN451664.1 | CPV48 | 1985 | dog | complete | - |
| 102 | MN451662.1 | CPV36 | 1984 | dog | complete | - |
| 103 | MN451661.1 | CPV35 | 1984 | dog | complete | - |
| 104 | MN451659.1 | CPV31 | 1983 | dog | complete | - |
| 105 | MN451658.1 | CPV30 | 1983 | dog | complete | - |
| 106 | MN451657.1 | CPV29 | 1983 | dog | complete | - |
| 107 | MN451655.1 | CPV12 | 1978 | dog | complete | - |
| 108 | MN451654.1 | CPV9 | 1979 | dog | complete | - |
| 109 | MN451653.1 | CPV6_1979 | 1979 | dog | complete | - |
| 110 | MN451656.1 | CPV27 | 1983 | dog | complete | - |
| 111 | MF805798.1 | Canine/China/10/2016 | 2016 | canine | complete | - |
| 112 | MF805797.1 | Canine/China/09/2016 | 2016 | canine | complete | - |
| 113 | MF805796.1 | Canine/China/08/2016 | 2016 | canine | complete | - |
| 114 | MF805795.1 | Canine/China/07/2016 | 2016 | canine | complete | - |
| 115 | MF805794.1 | Canine/China/06/2016 | 2016 | canine | complete | - |
| 116 | MF805793.1 | Canine/China/05/2016 | 2016 | canine | complete | - |
| 117 | MF805792.1 | Canine/China/04/2016 | 2016 | canine | complete | - |
| 118 | MF805791.1 | Canine/China/03/2016 | 2016 | canine | complete | - |
| 119 | MF805790.1 | Canine/China/02/2016 | 2016 | canine | complete | - |
| 120 | MF805789.1 | Canine/China/01/2016 | 2016 | canine | complete | - |
| 121 | MH711902.1 | CU21 | 2016 | cat | complete | 2c |
| 122 | MH711894.1 | CU24 | 2016 | cat | complete | 2c |
| 123 | MH476593.1 | Canine/China/24/2017 | 2017 | canine | complete | 2a |
| 124 | MH476592.1 | Canine/China/23/2017 | 2017 | canine | complete | 2c |
| 125 | MH476591.1 | Canine/China/22/2017 | 2017 | canine | complete | 2a |
| 126 | MH476590.1 | Canine/China/21/2017 | 2017 | canine | complete | 2a |
| 127 | MH660909.1 | 5 MGL | 2017 | dog | complete | 2c |
| 128 | MH545963.1 | TN/CPV2a/2018 | 2018 | dog | complete | 2a |
| 129 | MH106700.1 | CPV-BJL3 | 2016 | dog | complete | - |
| 130 | MF457594.1 | OH20219 | 2015 | canine | complete | - |
| 131 | MG763189.1 | CPV-L | 2014 | canine | complete | - |
| 132 | MF069444.1 | CPV/Raccoon/RC20/BC_2016 | 2016 | raccoon | complete | - |
| 133 | MF069443.1 | CPV/Raccoon/RC19/BC_2016 | 2016 | raccoon | complete | - |
| 134 | JQ268284.1 | CPV-LZ2 | 2011 | dog | complete | 2b |
| 135 | KY073269.1 | UFMT | 2015 | dog | complete | 2c |
| 136 | KM457143.1 | UY364 | 2011 | dog | complete | 2a |
| 137 | KM457142.1 | UY370c | 2011 | dog | complete | 2c |
| 138 | KM457140.1 | UY365 | 2011 | dog | complete | 2a |
| 139 | KM457139.1 | recUY364 | 2011 | dog | complete | 2a |
| 140 | KM457136.1 | UY315 | 2011 | dog | complete | 2a |
| 141 | KM457135.1 | UY306 | 2011 | dog | complete | 2a |
| 142 | KM457132.1 | UY245 | 2010 | dog | complete | 2a |
| 143 | KM457121.1 | UY247 | 2010 | dog | complete | 2c |
| 144 | JQ686671.1 | Guangxi/CPV-2a | 2011 | canine | complete | 2a |
| 145 | EU310373.2 | cpv/nj01/06 | 2006 | unknown | complete | 2a |
| 146 | MK144546.1 | K01708-3 | 2017 | canine | complete | 2a |
| 147 | KX774252.1 | Bel2015-02 | 2015 | dog | complete | 2b |
| 148 | KX774251.1 | Bel2015-01 | 2015 | dog | complete | 2b |
| 149 | KX774250.1 | Bel2016-01 | 2016 | dog | complete | 2b |
| 150 | MK306290.1 | CPV2c_2 | 2017 | dog | complete | 2c |
| 151 | LC214970.1 | CPV/dog/HCM/22/2013 | 2013 | dog | complete | 2a |
| 152 | MK208969.1 | RDPV/17/1 | 2017 | Raccoon dog | NS | - |
| 153 | KX434460.1 | CPV_IZSSI_52238_12 | 2012 | dog | No Capsid | 2c |
| 154 | MF177285.1 | UY269 | 2010 | dog | complete | 2c |
| 155 | MF177280.1 | E6 | 2011 | dog | complete | 2b |
| 156 | MF177274.1 | E31 | 2011 | dog | complete | 2c |
| 157 | MF177262.1 | Py1 | 2009 | dog | complete | 2c |
| 158 | MF177249.1 | Arg71 | 2010 | dog | complete | 2c |
| 159 | MF177240.1 | 189-02 | 2002 | dog | complete | 2c |
| 160 | MF177239.1 | 288-01 | 2001 | dog | complete | 2c |
| 161 | MF177234.1 | 347-03 | 2003 | dog | complete | 2c |
| 162 | MF177232.1 | 201-98 | 1998 | dog | complete | 2b |
| 163 | MF177230.1 | 57-10 | 2010 | dog | complete | 2c |
| 164 | MF996337.1 | RDPV-DP3 | 2016 | raccoon dog | NS | - |
| 165 | MF510158.1 | CPV_IZSSI_41113_c1_16 | 2016 | dog | complete | 2c |
| 166 | JF414819.1 | Arg35 | 2008 | dog | No NS | 2c |
| 167 | JF414817.1 | Arg5 | 2003 | dog | No NS | 2b |
| 168 | JF346754 | Arg9 | 2003 | dog | No NS | 2a |
| 169 | KM236568 | Choco | 2012 | dog | No NS | 2c |
| 170 | JF414818.1 | Arg32 | 2008 | dog | No NS | 2c |
| 171 | DQ340409.1 | BR183-85 | 1985 | dog | No NS | 2b |
| 172 | GU569944.1 | GZ0201 | 2002 | canine | No NS | 2b |
| 173 | MF467233.1 | CPV-HN1587 | 2015 | dog | No NS | 2b |
| 174 | KC196081.1 | M95 | 2007 | dog | No NS | 2c |
| 175 | JN867602.1 | CPV-2b/Dog/CA/148743/08 | 2008 | dog | No NS | 2b |
| 176 | KX425922.1 | KN-1/New CPV-2a/2015/Ind | 2015 | dog | No NS | 2a |
| 177 | MW653254.1 | CPV/18/Iran | 2020 | canin | capsid | - |
| 178 | LC570804.1 | 2017-11-KT-01 | 2017 | Asian small-clawed otter | capsid | - |
| 179 | MT270597.1 | BJ001 | 2019 | canine | capsid | 2c |
| 180 | FJ005261.1 | G162/97 | 1997 | Canis familiaris | capsid | 2b |
| 181 | DQ340410.1 | BR315-86 | 1986 | dog | capsid | 2a |
| 182 | GQ379044.1 | KU143_09 | 2009 | canine | capsid | - |
| 183 | AY742933.1 | CPV-339 | 1993 | canine | complete | - |
| 184 | KT275255.1 | PT238/14 | 2014 | dog | capsid | 2c |
| 185 | JF414821.1 | Arg48 | 2009 | canine | capsid | - |
| 186 | FJ005203.1 | G52-9/2-98 | 1998 | canine | capsid | 2c |
| 187 | GQ865519.1 | GR09/09 | 2009 | unknown | capsid | 2c |
| 188 | KF539797.1 | H-11 | 2012 | unknown | capsid | - |
| 189 | MN810918.1 | CC-3 | 2018 | canine | capsid | 2c |
| 190 | MN810914.1 | CC-8 | 2018 | canine | capsid | 2c |
| 191 | MN810887.1 | CC-721 | 2018 | canine | capsid | 2b |
| 192 | MN810885.1 | CC-820 | 2018 | canine | capsid | 2c |
| 193 | MN810882.1 | LY-2 | 2017 | canine | capsid | 2a |
| 194 | MN810880.1 | LY-4 | 2019 | canine | capsid | 2a |
| 195 | MN810879.1 | LY-5 | 2019 | canine | capsid | 2c |
| 196 | MT029326.1 | RDPV-2016-15/CPV-2 | 2016 | raccoon dog | capsid | - |
| 197 | MT029324.1 | RDPV-2016-10/CPV-2 | 2016 | raccoon dog | capsid | - |
| 198 | MT029322.1 | RDPV-2016-4/CPV-2 | 2016 | raccoon dog | capsid | - |
| 199 | MN473467.1 | CPV-NX05 | 2019 | dog | capsid | - |
| 200 | MN473466.1 | CPV-NX04 | 2019 | dog | capsid | - |
| 201 | MN473464.1 | CPV-NX02 | 2019 | dog | capsid | - |
| 202 | MN528598.1 | CPV_6064_AUS_NW_NSW_2019 | 2019 | dog | capsid | - |
| 203 | MN528596.1 | CPV_Ar7_AUS_Syd_2017 | 2017 | dog | capsid | - |
| 204 | MN259063.1 | CPV_Ar6_AUS_QLD_06/2018 | 2018 | dog | capsid | - |
| 205 | MN259061.1 | CPV_Ar2_AUS_Syd_2017 | 2017 | dog | capsid | - |
| 206 | MN119620.1 | CH-ZJ-D16 | 2019 | dog | capsid | 2c |
| 207 | MN119616.1 | CH-ZJ-D12 | 2019 | dog | capsid | 2c |
| 208 | MN119612.1 | CH-ZJ-D8 | 2018 | dog | capsid | 2c |
| 209 | MN119610.1 | CH-ZJ-D6 | 2018 | canine | capsid | 2c |
| 210 | MN119604.1 | CH-HN-D29 | 2019 | canine | capsid | 2c |
| 211 | MN119599.1 | CH-HN-D24 | 2019 | canine | capsid | 2c |
| 212 | MN119593.1 | CH-HN-D18 | 2019 | canine | capsid | 2b |
| 213 | MN119581.1 | CH-HN-D6 | 2018 | canine | capsid | 2a |
| 214 | MN119575.1 | CH-AH-D16 | 2019 | canine | capsid | 2b |
| 215 | MK344470.1 | SV731/15 | 2015 | canine | capsid | - |
| 216 | MF467224.1 | CPV-ZJ1579 | 2015 | canine | capsid | 2a |
| 217 | KX618915.1 | CPV-Sing | 2015 | palm civets | complete | 2a |
| 218 | FJ869124.1 | KU4_08 | 2008 | canine | capsid | - |
| 219 | KF366250.1 | CPV/915-H | 2013 | dog | complete | 2a |
| 220 | KR611510.1 | CPV-LN-14-13 | 2013 | dog | capsid | 2b |
| 221 | KR611507.1 | CPV-LN-14-10 | 2014 | dog | capsid | 2a |
| 222 | KR611496.1 | CPV-JL-14-9 | 2014 | dog | capsid | 2a |
| 223 | KR611467.1 | CPV-HB-14-9 | 2014 | dog | capsid | 2b |
| 224 | KP893077.1 | QIACPV1403 | 2014 | dog | capsid | - |
| 225 | KP749837.1 | ANTU-1 | 2014 | dog | capsid | 2a |
| 226 | KP749839.1 | DUNHUA-1 | 2014 | dog | capsid | 2a |
| 227 | KM236573.1 | Tete | 2012 | dog | capsid | - |
| 228 | KM236572.1 | NNGag | 2012 | dog | capsid | 2a |
| 229 | KM236571.1 | Minimorci | 2012 | dog | capsid | - |
| 230 | KM236569.1 | Cuba | 2013 | dog | capsid | - |
| 231 | M24003.1 | CPV-15/2a | 1984 | dog | capsid | 2a |
| 232 | M23255.1 | CPV-d Cornell #320 | 1982 | dog | capsid | - |
| 233 | KC196114.1 | CPV-2a/M306 | 2011 | dog | capsid | 2a |
| 234 | U22192.1 | RD-80 | 1980 | Raccoon dog | capsid | - |
| 235 | MW653255.1 | CPV/8/Iran | 2020 | dog | complete | - |
| 236 | MK306289.1 | Korea CPV2c_1 | 2017 | dog | complete | - |
| 237 | MH476586.1 | Canine/China/17/2017 | 2017 | dog | complete | 2a |
| 238 | MH476583.1 | Canine/China/14/2017 | 2017 | dog | complete | 2c |
| 239 | MH106698.1 | CPV-BJL1 | 2015 | dog | complete | - |
| 240 | MF510157.1 | CPV_IZSSI_2743_17 | 2017 | dog | complete | 2c |
| 241 | MF069442.1 | CPV/Raccoon/RC14/BC_2010 | 2010 | raccoon | complete | - |
| 242 | MH476589.1 | Canine/China/20/2017 | 2017 | canine | complete | 2a |
| 243 | MF134808.1 | CPV-BJ03/17 | 2017 | canine | complete | 2a |
| 244 | KP749874.1 | YANJI-2 | 2014 | canine | NS | 2b |
| 245 | KP749873.1 | YANJI-1 | 2014 | canine | NS | 2c |
| 246 | KP749872.1 | WANGQING-3 | 2014 | canine | NS | 2a |
| 247 | KP749871.1 | WANGQING-2 | 2014 | canine | Ns | 2b |
| 248 | KP749870.1 | WANGQING-1 | 2014 | canine | NS | 2c |
| 249 | KP749869.1 | TUMEN-2 | 2014 | canine | NS | 2a |
| 250 | KP749868.1 | TUMEN-1 | 2014 | canine | NS | 2a |
| 251 | KP749867.1 | LONGJING-2 | 2014 | canine | NS | 2a |
| 252 | KP749866.1 | LONGJING-1 | 2014 | canine | NS | 2b |
| 253 | KP749865.1 | HUNCHUN-3 | 2014 | canine | NS | 2a |
| 254 | KF638400.1 | s5 | 2010 | unknown | complete | 2a |
| 255 | JX660690.1 | SC02/2011 | 2011 | dog | complete | 2a |
| 256 | HQ883272.1 | BJ/2010 | 2010 | canine | NS | 2a |
| 257 | HQ883266.1 | Beijing | 2010 | canine | NS | 2a |
| 258 | EF011664.1 | China/CPV-2a | 2004 | dog | complete | 2a |
| 259 | MT165694.1 | NewCPV-2a/China/SC-16/2017 | 2017 | canine | complete | 2a |
| 260 | KX434459.1 | CPV_IZSSI_27692_1_11 | 2011 | canine | complete | 2c |
| 261 | EU659121.1 | CPV-411b .us.98 | 1998 | canine | complete | - |
| 262 | EU659119.1 | CPV-410.us.00 | 2000 | canine | complete | - |
| 263 | EU659120.1 | CPV-411a.us.98 | 1998 | canine | complete | 2b |
| 264 | EU659118.1 | CPV-13.us.81 | 1981 | dog | complete | 2a |
| 265 | EU659117.1 | CPV-6.us.80 | 1980 | dog | complete | - |
| 266 | EU659116.1 | CPV-5.us.79 | 1979 | dog | complete | - |
| 267 | AY742936.1 | CPV-395 | 1998 | dog | complete | - |
| 268 | AY742935.1 | CPV-U6 | 1995 | dog | complete | - |
| 269 | MF457593.1 | OH19098-2 | 2015 | dog | complete | - |

‘-‘indicates no available, NS- non structures

**>Complete genome of CPV (UPM-CPV15/P. tigris jacksoni) isolated from tigris jacksoni***

ACGGTTTGCCTCTAGATGATAGGCGGTTTGTGTGTTTAACTTGGGCGGGAAAAGGTGGCGGGCTAATTGTGGGCGTGGTTAAAGGTATAAAAGACAAACCATAGACCGTTACTGACATTCGCTTCTTGTCTTTGACAGAGTGAACCTCTCTTACTCTGACTAACCAACCATGTCTGGCAACCAGTATACTGAGGAAGTTATGGAGGGAGTAAATTGGTTAAAGAAACATGCAGAAAATGAAGCATTTTCGTTTGTTTTTAAATGTGACAACGTCCAACTAAATGGAAAGGATGTTCGCTGGAACAACTATACCAAACCAATTCAAAATGAAGAACTAACATCTTTAATTAGAGGAGCACAAACAGCAATGGATCAAACCGAAGAAGAAGAAATGGACTGGGAATCGGAAGTTGATAGTCTCGCCAAAAAGCAAGTACAAACTTTTGATGCATTAATTAAAAAATGTCTTTTTGAAGTCTTTGTTTCTAAAAATATAGAACCAAATGAATGTGTTTGGTTTATTCAACATGAATGGGGAAAAGATCAAGGCTGGCATTGTCATGTTTTACTTCATAGTAAGAACTTACAACAAGCAACTGGTAAATGGCTACGCAGACAAATGAATATGTATTGGAGTAGATGGTTGGTGACTCTTTGTTCGGTAAACTTAACACCAACTGAAAAGATTAAGCTCAGAGAAATTGCAGAAGATAGTGAATGGGTGACTATATTAACATACAGACATAAGCAAACAAAAAAAGACTATGTTAAAATGGTTCATTTTGGAAATATGATAGCATATTACTTTTTAACAAAGAAAAAAATTGTCCACATGACAAAAGAAAGTGGCTATTTTTTAAGTACTGATTCTGGTTGGAAATTTAACTTTATGAAGTATCAAGACAGACAAATTGTCAGCACACTTTACACTGAACAAATGAAACCAGAAACCGTTGAAACCACAGTGACGACCGCACAGGAAACAAAGCGCGGGAGAATTCAAACTAAAAAGGAAGTGTCAATCAAATGTACTTTGCGGGACTTGGTTAGTAAAAGAGTAACATCACCTGAAGACTGGATGATGTTACAACCAGATAGTTATATTGAAATGATGGCACACCCAGGAGGTGAAAATCTTTTAAAAAATACACTTGAAATTTGTACTTTGACTTTACCAGAACAAAAAAACGGCTTTGGATTTATTCTTGGAAAAGCGGATAATACTAAACTAACTAACTTTGATCTTGCAAATTCTAGAACATGTCAAATTTTTAGAATGCACGGATGGAATTGGATTAAAGTTTGTCACGCTATAGCATGTGTTTTAAATAGACAAGGTGGTAAAAGAAATACAGTTCTTTTTCATGGACCAGCAAGTACAGGAAAATCTATCATTGCTCAAGCCATAGCACAAGCTGTGGGTAATGTTGGTTGTTATAATGCAGCAAATGTAAATTTTCCATTTAATGACTGTACCAATAAAAATTTAATTTGGATTGAAGAAGCTGGTAACTTTGGTCAACAAGTTAATCAATTTAAAGCAATTTGTTCTGGACAAACAATTAGAATTGATCAAAAAGGTAAAGGAAGTAAGCAAATTGAACCAACTCCAGTAATTATGACAACTAATGAAAATATAACAATTGTGAGAATTGGATGTGAAGAAAGACCTGAACATACACAACCAATAAGAGACAGAATGTTGAACATTAAGTTAGTATGTAAGCTTCCAGGAGACTTTGGTTTGGTTGATAAAGAAGAATGGCCTTTAATATGTGCATGGTTAGTTAAACATGGTTATGAATCAACCATGGCTAACTATACACATCATTGGGGAAAAGTACCAGAATGGGATGAAAACTGGGCGGAGCCTAAAATACAAGAAGGTATAAATTCACCAGGTTGCAAAGACTTAGAGACACAAGCGGCAAGCAATCCTCAGAGTCAAGACCAAGTTCTAACTCCTCTGACTCCGGACGTAGTGGACCTTGCACTGGAACCGTGGAGTACTCCAGATACGCCTATTGCAGAAACTGCAAATCAACAATCAAACCAACTTGGCGTTACTCACAAAGACGTGCAAGCGAGTCCGACGTGGTCCGAAATAGAGGCAGACCTGAGAGCCATCTTTACTTCTGAACAATTGGAAGAAGATTTTCGAGACGGCTTGGATTAAGGTACGATGGCACCTCCGGCAAAGAGAGCCAGGAGAGGTAAGGGTGTGTTAGTAAATTGGGGGGGAGAGGGAAAAAATTTTAAAACTTGTGCCTCCAGGTTATAAATATCTTGGGCCTGGGAACAGTCTTGACCAAGGAGAACCAACTAACCCTTCTGACGCCGCTGCAAAAGAACACGACGAAGCTTACGCTGCTTATCTTCGCTCTGGTAAAAACCCATACTTATATTTCTCGCCAGCAGATCAACGCTTTATAGATCAAACTAAGGACGCTAAAGATTGGGGGGGGAAAATAGGACATTATTTTTTTAGAGCTAAAAAGGCAATTGCTCCAGTATTAACTGATACACCAGATCATCCATCAACATCAAGACCAACAAAACCAACTAAAAAAAATAAACCACCACCTCATATTTTCATCAATCCTGCAAAAAAAAAAAAAGCGGGGGGGGGACAAGTAAAAAGAGACAATCTTGCACCCATGAGTGATGGAGCAGTTCAACCAGACGGGGGTCAACCTGCTGTCAAAAATGAAAGAGCTACAGGATCTGGGAACGGGTCTGGAGGCGGGGGTGGTGGTGGTTCTGGGGGTGTGGGGATTTCTACGGGTACTTTCAATAATCAGACGGAATTTAAATTTTTGGAAAACGGATGGGTGGAAATCACAGCAAACTCAAGCAGACTTGTACATTTAAATATGCCAGAAAGTGAAAATTATAGAAGAGTGGTTGTAAATAATATGGATAAAACTGCAGTTAACGGAAACATGGCTTTAGATGATACTCATGCACAAATTGTAACACCTTGGTCATTGGTTGATGCAAATGCTTGGGGAGTTTGGTTTAATCCAGGAGATTGGCAACTAATTGTTAATACTATGAGTGAGTTGCATTTAGTTAGTTTTGAACAAGAAATTTTTAATGTTGTTTTAAAGACTGTTTCAGAATCTGCTACTCAGCCACCAACTAAAGTTTATAATAATGATTTAACTGCATCATTGATGGTTGCATTAGATAGAAATAATACTATGCCATTTACTCCAGCAGCTATGAGATCTGAGACATTGGGTTTTTATCCATGGAAACCAACCATACCAACTCCATGGAGATATTATTTTCAATGGGATAGAACATTAATACCATCTCATACTGAAACTAGTGGCACCCCAACAAATATATACCATGGTACAGATCCAGATGATGTTCAATTTTATACTATTGAAAATTCTGTGCCAGTACACTTACTAAGAACAGGTGATGAATTTGCTACAGGAACATTTTTTTTTGATTGTAAACCATGTAGACTAACACATACATGGCAAACAAATAGAGCATTGGGCTTACCACCATTTCTAAATTCTTTGCCTCAATCTGAAGGAGCTACTAACTTTGGTGATATAGGAGTTCAACAAGATAAAAGACGTGGTGTAACTCAAATGGGAAATACAAACTATATTACTGAAGCTACTATTATGAGACCAGCTGAGGTTGGTTATAGTGCACCATATTATTCTTTTGAGGCGTCTACACAAGGGCCATTTAAAACACCTATTGCAGCAGGACGGGGGGGAGCGCAAACAGATGAAAATCAAGCAGCAGATGGTAATCCAAGATATGCATTTGGTAGACAACATGGTCAAAAAACTACCACAACAGGAGAAACACCTGAGAGATTTACATATATAGCACATCAAGATACAGGAAGATATCCAGAAGGAGATTGGATTCAAAATATTAACTTTAACCTTCCTGTAACAAATGATAATGTATTGCTACCAACAGATCCAATTGGAGGTAAAACAGGAATTAACTATACTAATATATTTAATACTTATGGTCCTTTAACTGCATTAAATAATGTACCACCAGTTTATCCAAATGGTCAAATTTGGGATAAAGAATTTGATACTGACTTAAAACCAAGACTTCATGTAAATGCACCATTTGTTTGTCAAAATAATTGTCCTGGTCAATTATTTGTAAAAGTTGCGCCTAATTTAACAAATGAATATGATCCTGATGCATCTGCTAATATGTCAAGAATTGTAACTTACTCAGATTTTTGGTGGAAAGGTAAATTAGTATTTAAAGCTAAACTAAGAGCCTCTCATACTTGGAATCCAATTCAACAAATGAGTATTAATGTAGATAACCAATTTAACTATGTACCAAGTAATATTGGAGGTATGAAAATTGTATATGAAAAATCTCAACTAGCACCTAGAAAATTATATTAACATACTTACTATGTTTTTATGTTTATTACATATCAACTAGCACCTAGAAAATTATATTAATATACTTACTATGTTTTTAGGTTTATAACATATTATTTTAAGATTAATTAAATTACAGCATAGAAATATTGTACTTGTATTTGAAATAGGATTTAGAAGGTTTGTTAGATGGTATACAATAACTGTTAGAAATAGAAGAACTTTTAGATCAAAGTTAGGAATTTTGTTTTAAAAAATGTATTGTAAACCATTAATGTAGTTGTTAGGTGGGGGGTGGGTTTTGTTTGTTGGCCAAGTAGG

*Sequence has been submitted to GenBank by removing the sentences of awaiting for accession no. This is because accession number has been granted by NCBI, MW380384.
